# Self-medication pattern among medical students in Middle Delta, Egypt

**DOI:** 10.1186/s12909-025-06678-x

**Published:** 2025-01-21

**Authors:** Nadira Mansour Hassan, Shimaa Mohamed Mohamed Koabar

**Affiliations:** Public Health and Community Medicine Department, Faculty of Medicine, Tanta University, Tanta, Egypt

**Keywords:** Medical students, Self-medication, Pattern, Analgesics, Antibiotics

## Abstract

**Background:**

Self- medication leading to wastage of limited resources in developing countries, prolonged suffering, increase resistance to drugs and may result in significant medical complications such as adverse drug responses and dependence. Self-medication is extensively used by health professionals. Undergraduate medical students as being the future physicians representing a main pillar in health care system thus have special significance. This study aimed to estimate the prevalence of self-medication and identify its pattern among medical students in Tanta university, Egypt.

**Methods:**

A four-month cross-sectional study was carried out among Egyptian undergraduate medical students at Tanta University, from first to final year. (November 2023 – February 2024). The students were chosen using a two-stage cluster sampling method, and data on sociodemographic and self-medication patterns were collected using a predesigned semi-structured self-administered questionnaire.

**Results:**

The prevalence of self-medication was 71%. The key determinants of self-medication was students’ medical knowledge from self- experience and studies (55.9%). Headache was a common complaint for self-medication (80.4%). Majority of them (88.3%) use pharmaceutical products where analgesics lies on top (92.4%). The degree of popularity of the medicine was the primary reason for drug selection (52.5%) and recommendation of the pharmacist was the main determinate of selecting type of drug (43.6%). However, 30.2% experienced side effects, of which 50% went to private physician and 33.5% stopped taking their medications. Half of the students took antibiotics for self-medication.

**Conclusions:**

Self-medication is a common practice among medical students. where headache was the common symptom and the most commonly utilized medications for self-medication were analgesics. There is a need to augment the value of diagnosis, awareness and seriousness of this practice.

**Supplementary Information:**

The online version contains supplementary material available at 10.1186/s12909-025-06678-x.

## Background

The use of pharmaceuticals by individuals to address self-recognized diseases or symptoms is known as self-medication (SM). Self-medication entails obtaining drugs without a prescription, resubmitting previous prescriptions to purchase medicines, exchanging medicines with family members or members of one's social network, or using leftover medication stored in the household. It also encompasses the use of a wide spectrum of complementary and alternative medicine, such as herbal medicine (herbal formulations and herb products), nutritional supplements, and home remedies [1, 2].

Self-medication is frequent among medical students as a result of the knowledge they have, lack of understanding of the adverse effects of using medications, having easy access to the internet, and higher media coverage of associated health issues [3, 4].

Self- medication leads to wastage of limited resources in developing countries, prolonged suffering, increased drug resistance, drug reactions and dependence. Also, it lead to masking of diagnosis and sometimes chronicity of diseases [5, 6]. Self-medication using antibiotics poses a high risk of developing drug-resistant bacteria and, as a result, eventual treatment failure [7].

SM has recently grown in popularity throughout the world, including both developed and developing countries. Multiple research investigations have found that self-medication is a widespread issue among medical students [8] During the previous two decades, the prevalence of prescription misuse has steadily climbed in Egypt. Medication misuse is becoming more common to be as high as 86.4% [9–11].

### Rationale

Medical students as being the future physicians responsible for drug prescription and representing a main pillar in health care system so, it is quite significant to identify self-medication prevalence and its patterns among them to go into depth with the problem.

### Aim

The purpose of the present research was to estimate prevalence of self-medication and identifying its pattern among Tanta University medical students, Middle Delta, Egypt.

## Methods

### Study design, setting and participants

A descriptive cross-sectional study was carried out for a period of four months (November 2023 – February 2024). The target population was Egyptian undergraduate medical students from first to final year in Tanta University, this is a Governmental University in the Middle Delta region that recruits students from lower Egyptian governorates.

### Inclusion criteria

Inclusion criteria for the study was medical students at Tanta faculty of medicine from -first to fifth grade with good mental health and who didn't mind participating in the study.

### Exclusion criteria

Exclusion criteria include those who were absent or have serious illness /or hospitalized during the period of data collection.

### Sample type and size

The sample type was two stage cluster random sample. The target population was all grades of medical students at faculty of medicine in Tanta University from 1st to 5th grade with a total number of (5736). The sample size was calculated using the center for Disease Control and Prevention, Atlanta, Georgia, USA Epi‐Info 7.2.3.0 software statistical package. The incidence of self-medication practice among undergraduate medical students was assessed to be 78% according to a previous study [12], and using a 95% confidence interval and a precision of 5%, the minimum required sample size was determined to be 250 persons. To compensate incompleteness of the data and non-responsiveness, 10% of the minimum required sample size (25 students) was added. So, the total number was 275 students. The number of students from first to fifth grade was (1482,1208,1229,1016,801) respectively. We calculate the required sample size of each grade divided by their weight (each grade/total student number from all grades *sample size). The sample size of students from first to fifth grade was 71,58,59,49 and 38 respectively. As each grade divided into rounds and each round in each grade was enrolled nearly 120 students so at the first stage of the cluster sampling, one round was randomly selected from each grade and at the second stage: the required number of students was randomly selected using the faculty coding number of students. Only 252 students respond with complete questionnaires with a response rate of 91.6%

### Study tool

Data were gathered using a predesigned semi structured self-administrated questionnaire that consists of 21 questions about self-medication. The questionnaire was developed by the investigators after reviewing past literatures [6, 8, 11, 13] and experts of two departments (Public Health and Community Medicine, and pharmacology departments) reviewed the questionnaire to assess its validity. The questionnaire contained two parts:

- **Part 1** included the socio demographic characteristics of the participants as age, sex, grade, marital state, parent education, number of family members and family income.
- **Part 2** consisted of questions about self-medication. It additionally involved questions about the underlying reasons for self-medication**.** types of self-medication (drugs, nutritional supplements, home remedies or herbal preparations) and its pattern, the complaint for which self- medication was practiced, side effects resulting and chronic diseases if present. And also questions about self-medication with antibiotics.

#### Validity of the questionnaire

Content validity was assed according to the following procedure:1) Preparing content validation form (2) Selecting a review panel of experts (five experts of two departments (Public Health and Community Medicine, and pharmacology departments) reviewed the questionnaire) (3) Conducting content validation (4) Reviewing domain and items (5) Providing score on each item (6) Calculating content validity index (CVI).

Each expert was asked to rate all the questions on a scale ranging from “not relevant = 1,” “slightly relevant = 2,” “relevant, but needs minor modifications = 3,” to “completely relevant = 4.” The CVI for each item was calculated by dividing the number of experts who gave a rating of 3 or 4 by the total number of experts. Items with a CVI score ≥ 0.8 were considered sufficient. The initial questionnaire was 23 items, two items were excluded and the remaining 21 item were either rating 3 or 4 by all experts those rating 3 had some minor modifications so CVI was 21/21 = 1 which was acceptable as satisfactory for content validity [14]. According to experts’ opinions all questions were matching the objectives of the study and the target population, understandable and comprehensive. To ensure the questionnaire validity and reliability; pilot study of 20 medical students (not included at the study) were recruited and asked to fulfill the questionnaire. The students agreed that the content was appropriate, the questions were clear and no changes were required. They fulfill the questionnaire at 5 to 10 mints. Cronbach’s alpha was 0.86 which represented adequate internal consistency.

#### Ethical consideration

Formal consent was obtained from participants and Individuals who refused to participate were excluded from participating. The goal and procedure of the study, as well as the benefits of sharing it, were explained to participants. Data was collected anonymously and not used for any other purpose than scientific research. Confidentiality and privacy were maintained throughout the study, The study's ethical considerations were guided by the Tanta Faculty of medicine's research ethics committee (approval number 36264PR82/2/23).

### Data collection tool and method

The data was collected from the students before starting their rounds. The purpose of the study and details on the questionnaire was explained to the respondents. After a formal consent and assuring about the confidentiality of their information, the questionnaires were distributed to those students that were selected randomly, asked to fill it up by themselves,

### Statistical analysis

The data were analyzed using the Statistical Package for the Social Sciences" SPSS 22.0 software (IBM Microsoft). Qualitative variables were prescribed using numbers and percentages. Also, chi-square and Montecarlo tests were used, and numerical variables were expressed as means and standard deviations.

## Results

Table 1: demonstrated sociodemographic characteristics of the studied participants, and its relationship with self-medication status. The sample included 252 students, 60% were females (151 students). About two- thirds of the sample was from rural areas (152 students). The participants were from the first to five grades each grade shared by 25.4%, 20.6%, 21.4%, 17.9% and 14.7% respectively in the study. Around two-thirds of the participants' fathers and mothers completed university education (65.5%, and 61.1% respectively). About two- thirds of the participants' family income was just enough (65.1%). There was no significant association between self-medication status and sociodemographic characteristics.

**Table 1:** Sociodemographic Characteristics of the studied medical students and their relationship with self-medication status

Variable	Self-medication				Total		Significance test *p*-value
	Yes		No		No	%	
	N 179	% 71	N 73	% 29	N 252	% 100	
**Sex**							
-Male	75	74.3	26	25.7	101	40.0	*X*^2^ = 0.852
-Female	104	68.9	47	31.1	151	60.0	*p* = 0.356
**Grade:**							
1st	50	78.1	14	21.9	64	25.4	MC = 6.559 *p* = 0.161
2nd	31	59.6	21	40.4	52	20.6	
3rd	36	66.7	18	33.3	54	21.4	
4th	35	77.8	10	22.2	45	17.9	
5th	27	73.0	10	27.0	37	14.7	
**Marital Status**							
-Single	169	70.1	72	29.9	241	95.6	*X*^2^ = 2.374 *p* = 0.305
-Others	10	90.9	1	9.1	11	4.5	
**Residence**							
-Rural	102	67.1	50	32.9	152	60.3	*X*^2^ = 2.870 *p* = 0.090
-Urban	77	77.0	23	23.0	100	39.7	
**Family income**							
Not enough	13	81.2	3	18.8	16	6.3	MC = 5.155 *p* = 0.076
Just enough	122	74.4	42	25.6	164	65.1	
Enough and saving	44	61.1	28	38.9	72	28.6	
**Father education**							
Illiterate Basic education (Primary, preparatory) Secondary education High education	1 23 44 111	100 65.7 86.3 67.3	0 12 7 57	0.0 34.3 13.7 32.7	1 35 51 165	0.04 13.9 20.2 65.5	MC = 7.781 *p* = 0.037
**Mother**							
Illiterate Basic education (Primary, preparatory) Secondary education High education	6 25 43 105	54.5 71.4 84.3 68.2	5 10 9 49	45.5 28.6 15.7 31.8	11 35 52 154	4.4 13.9 20.6 61.1	MC = 6.944^a^ *p* = 0.139
**Age (17–23 years) Mean ± SD** (20.0 ± 1.3)							

Table 2 showed prevalence, and determinants affecting self-medication selection: the prevalence of self-medication among the studied group was 179(71%) The prevalence of self-medication with antibiotics was 128(50.8%)., Regarding determinants, medical knowledge from self- experience and studies was the main reported reason100 (55.9%), Pharmacist advice 78(43.6%) then having medicines of family members, peers, friends 74(41.3%). An old prescription 63(35.2%) and saving time 60(33.5%).

**Table 2:** Prevalence and determinants for self-medication^a^

Item	*N* = 252	%
Prevalence of self-medication	179	71.0
Self-medication with antibiotic	128	50.8
Taking medication for chronic disease	34	13.5
Determinants of self- medication (*n* = 179)		
- Medical knowledge from self-experience and studies	100	55.9
- Pharmacist advice	78	43.6
- Used by peers – friends / family members	74	41.3
- An old prescription from a physician	63	35.2
- Saves time	60	33.5
- Advertisement	28	15.6
- Doctor / clinic far from home	27	15.1
- High fees of doctor	21	11.7
**-** Others	12	6.7
Factors affecting the type of selected-self-medication:		
-The commonest used medication	94	52.5
- Form of medicine (tablets)	74	41.3
- Price	28	15.6
**-** Pharmaceutical company	26	14.5

^a^multiple answers were allowed

The main factors affecting selecting type of self-medication were; the most commonly used medication among population 94(52.2%), followed by the form of medicine; tablets 74(41.3%) and its price 28(15.6%).

Table 3: revealed types of self-medication and type of pharmaceutical product; where the most common type of self- medication was pharmaceutical products (88.3%), followed by nutritional supplements (30.7%), complementary and alternative medicine (16.2%) and home remedies (13.9%). Analgesics lies on top of used pharmaceutical product by a percent of 92.4% then antipyretics by a percent of 43%.

**Table 3:** Types of self-medication and type of pharmaceutical product^a^

Item	*N* = 179	%
**Type of self-medication:**^**a**^		
- Pharmaceutical products	158	88.3
- Nutritional supplements	50	30.7
- Home remedies (ginger, mint…etc.)	29	16.2
- Complementary and alternative medicine (Such as herbal medicines (Herbs or herbal preparations)	25	13.9
**Type of pharmaceutical product (*n* = 158)**		
- Analgesics	146	92.4
- Antipyretics	68	43.0
- NSAIDs (Non-Steroidal Anti Inflammatory Drugs)	41	25.9
- Antihistamines	18	11.4
- Hypnotics	7	4.4
- Others	17	10.8

^a^multiple answers were allowed

Table 4 revealed the frequency of complaint during the last three months that led to self-medication, the most common complaints were headache (80.4%), followed by pain (79.9%), and then respiratory and gastrointestinal (73.2% and 60.9%) respectively.

**Table 4:** Frequency of complaint that led to self-medication at the last 3 months^a^

System	Complaint	No	%
Head	Headache	144	80.4
Pain	Body pains, muscle, joint, dental, ear and menstrual problems	134	79.9
Respiratory	Cough, running nose, fever, and bronchial asthma	131	73.2
GIT complaints	Vomiting, diarrhoea, nausea, and difficulty in swallowing	109	60.9
Skin	Hair fall, skin diseases with rash, dandruff, and wounds	72	40.2
Nervous	Epilepsy, migraine, and faints	40	22.3
Endocrine	Diabetes and hypertension	10	5.6
Others	Reproductive and urinary	13	7.3

^a^multiple answers were allowed

Table 5 presented sources of used medication, experience of side effects, and response to side effects. Most of participants obtained their medications from the pharmacy (59.8%) followed by primary health care center (32.4%). On the other hand, 30.2% experienced side effects and out of them 50% went to a private physician and about 33.5% stopped taking their medications.

**Table 5:** Sources of used medication, experience of side effects, and response to side effect

Item	No	%
**-Source of used medication:**^**a**^		
- Pharmacy	107	59.8
- Primary health care centre	58	32.4
- Friends / family	18	10.1
- Online shopping	18	10.1
- Others	6	3.4
**Experiencing side effect of self-medication**	54	30.2
**Response to side effects:**^**a**^**n-54**		
- Go to private physician	27	50
- Stop taking medication	18	33.5
-Go to pharmacy	16	29.6
- Go to primary health care centre	11	20.4
- Others	3	5.6

^a^multiple answers was allowed

## Discussion

According to the present study, seventy-one percent (71%) of the studied medical students were practicing self-medication, where headache was the most common complaint. Analgesics were the prevalent pharmaceuticals drugs used by the participants. They were used self-medication depending mainly on their medical knowledge from self- experience and studies. The recommendation by the pharmacist was an important determinant for self-medication Since the pharmacy was the main supplier for self-medication. On the other hand, about one-third, experienced side effects with self-medication, and about half of them resorted to a private physician. More than half of respondents reported self-medication with antibiotics.

### Prevalence and knowledge regarding self-medication

The prevalence of self-medication practice in the present study was in agreement with the findings of Ali et al.(2024) in Egypt, who reported that only 62.80% practiced SM, also Younis et al.(2022) in Egypt, who reported that about three-quarters of the students (74.6%) practice self-medication [11, 15]. This may be attributed to similarity of the target population(medical students) and their prior knowledge and experience from medical educational courses, ability to read research papers and books about various medications and understand the labels of the consumed medicine. Furthermore, living on the campus may alter students' views and make them more vulnerable to self-medication in some situations. Some previous studies showed lower prevalence rates Malli et al. (2023) in Saudia Arabia, who reported that the prevalence of self-medication was (55.9) % [16], also in Ethiopia (58.5%), Jordan (42.5%), Bahrain (44.8%) and Western Nepal (59%) [17–20], others documented higher ones as revealed in Pakistan (83%), Bangladesh (88.3%) and Palestine (98%) [21–23]. In Palestine the rate was very high, and this may be due to the siege and shortage of health services.

### Indications, attitudes, and categories for self-medication

At the present study headache (80.4%) and pain (79.9%), at various sites of the body were the common illness stated by vast majority of the survey participants who were using self-medication. This was in consistence with the findings of previous studies in Malli et al. (2023) in Saudia Arabia, (94%), Turan et al. (2024) in turkey (83.1%)), India (64.7%) [16, 24, 25], Iran (85.5%) and Iraq (92%) [26, 27]. The second cause of self-medication was respiratory problems. Helal et al. reported a rate of 70.1% [8] Haroun and Al-kayali (80.6%) [28] in contrast with Ali et al. (2024) in Egypt, who reported that the most common complaint for self-medication was cardiovascular diseases (46%) [11].

### Reasons for using self-medication

Medical knowledge from self- experience and studies was on top of major determinants of self-medication for participants of this study (55.9%), followed by pharmacist advice (43.6%) where, most of them use medications which were recommended by the pharmacist. Also, previous use with peers/or family members (41.3%), old prescription from a physician (35.2%) and time savings (33.5%) were the primary motivators for self-medication as reported by participants. This was inconsistent with the findings with Ali et al. (2024) in Egypt, who reported The pharmacist’s recommendation was the source of SM for 53.61% while 31.53% used old medications at home [11].

Turan et al. (2024), who reported that 64.6% of students practice self-medication because they had experienced a similar illness before, also Malli et al. (2023) in Saudia Arabia, who reported that (72.2%) of participants said their past experience with the same illness justified using the same medicine without renewing it. 69.6% of students said self-medication reduces doctor visits, [16, 24].

Parulekar et al. (2016) in their systematic review where they documented that previous experience with the symptoms and medicine together with time saving cost-effectiveness were the key determinants of self-medication [29].

Conversely, in the research conducted by also Tohan et al. (2024) in Bangladesh, the convenience of self-medication in Bangladesh is attributed to the knowledge gained from previous prescriptions and the online availability of medical information. Those factors were reported as key drivers of self-medication [30]. More over in Ali et al. (2024) in Egypt reported that practiced SM used the old medications at home and refilled the previous prescriptions respectively. Malli et al. (2024) in Saudi Arabia reported that 77.8% of students believed that using self-medication alone was sufficient to treat mild illnesses, while 78.7% believed that time is saved by avoiding hospital visits for consultations. Turan et al. (2024) in turkey reported that, keeping medicines at home or in the dormitory for later use significantly increased the likelihood of SM [11, 16, 24] additionally in researches done by Ozdinc et al. and Pourreza et al. [31, 32], who reported that prior experience with the same medicine in comparable settings and attaining the intended outcome were the major factors of self-medication among persons.

### Places and forms of self-medication

As stated in the present research, the pharmacy was the main place for obtaining self-medication as reported by more than one-half of students (59.8%). The pharmacy is a regular, safe place to purchase pharmaceuticals in Egyptian society, and it is generally accessible. Egypt has no special limitations on distributing analgesics and antibiotics through community pharmacies. Such issues are exacerbated by the needless and unfavorable practice of community pharmacists. While some of them may have enough expertise to properly utilize antibiotics, the majority of them do not apply these regulations in clinical practice [11].

The finding was revealed in studies that were carried out in Ali et al. (2024) in Egypt [11] Ethiopia [17] and Syria, [28]. This pointed to a faulty system for drug distribution. Therefore, pharmacies that sell medications without a prescription or drug-related laws must be more seriously monitored to solve this problem.

On the other hand, analgesics were the prevalent pharmaceutical products used by the vast majority of studied students (92.4%). This was in agreement with Malli et al. (2024) in Saudi Arabia reported that (85.6%) of students used analgesics mainly for self-medication followed by antipyretic, Ali et al.(2024) in Egypt,reported painkillers were the most used by most participants (60.4%)followed by antibiotics(32%) used by one-third of participants. Analgesic and antibiotic misuse are well-established problems in Arab countries, especially Egypt. Turan et al. (2024) in turkey reported that The most frequently self-administered medications were analgesics (86.1%), common cold medicines (54.8%), vitamins (34.0%), and stomach medications [11, 16, 24]. Also these came in agreement with other studies such as Ezz et al. [33] at Ain Shams University in Egypt and other Arabic countries as Bahrain [19] and Palestine [23].

The growing practice of analgesic self-administration has been linked to drug tolerance, and dependency (with opioids), and may even hide the real diagnosis of the underlying illness [34, 35].

The current study revealed that half of medical students took self-medication in the form of antibiotics. indicates that increasing knowledge of Clinical Pharmacology and other clinical training may contribute to this rise This is in line with other studies in Iran (53%) and in Emirate (Abu Dhabi) (56%), [35, 36]. Higher than Turan et al. (2024) in turkey reported that the rate of using antibiotics without a prescription was (19%) among the students who participated in their study. As the sale of antibiotics without a prescription is prohibited in pharmacies in Turkey, it is likely that self-administered antibiotics are left over from previous treatments that were not fully completed [24].

Abuse of antibiotics is the main cause of bacterial resistance so intake must be appropriately regulated [37]. In May 2015, the World Health Organization supported the "Global Action Plan on Antimicrobial Resistance," a document that urges all nations to implement national antibiotic resistance policies within two years [38, 39]. The authors are optimistic that national level action plans will combat antibiotic resistance, combined with findings from existing and future studies, will assist individual countries in understanding the magnitude of antibiotic resistance in their geographic area and then taking coordinated, research-backed initiatives to control it [38, 39].

Regarding side effects with self-medication, about one third of respondents experienced side effects, half of them consulted private physicians. This was higher than Ezz et al. [33] who stated that only (4.8%) experienced side effects, and 82.3% of them thought that in the event of adverse effects, the assistance of a physician is essential. Also, Malli et al. (2023) in Saudi Arabia stated that most students (85.2%) agreed that self-medication might lead to unexpected reactions, about (92.0%) reported that overusing self-medication can result in drug resistance. Most subjects (94.6%) agreed that physicians’ help must be sought in cases of side effects [16].

A study in Kuwait reported that about 69% preferred consulting a doctor in case of adverse reactions [39].

### Limitations of the study

The study was a cross-sectional study with lack of causality. Also, it included students from a single medical university with lack of comparability in addition to the smaller number of students enrolled in the study. The study's reliance on self-reported data, which could result in errors because of recall bias or forgetfulness, is another drawback. Multicenter research and qualitative studies on the topic can offer additional insights into the reasons behind SM in medical students as well as ways to lessen it.

## Conclusion

Self-medication was widely used among the medical students. with the majority using it to manage headaches and pains. Analgesics were the main pharmaceutical products used to alleviate these complaints. Also, antibiotics were commonly used without prescription. The main determinants of this practice were previous medical knowledge and experience, pharmacist advice and usage by peers or family members in an attempt to save time and get quick relief.

### Recommendations

At the educational level; as a future health care provider, medical students should value the importance of diagnosis more than relief of symptoms like headaches and other pains all over the body. These complaints may be due to serious diseases and the pracice of self-medication may lead to temporary relief of symptoms and progress of the underlying disease.

At the level of pharmacy, monitoring prescription and volume of sales comparison particularly for antibiotics ought to be a standard feature of drug regulatory agencies' inspection and audit.

By applying the current technology and user-friendly nature of cell phones and cell phone applications, apps might be built to convey prescribed medicines from physician to pharmacist depending on which dispensing occurs to consumers. This strategy would limit prescription reuse while also assisting in data storage, which could be supervised and inspected by drug regulatory agencies.

## Supplementary Information

Supplementary Material 1.

## Data Availability

Data is provided within supplementary information files.

## Abbreviation

SM: Self-medication
