# Supplementary material for: Self-medication pattern among medical students in Middle Delta, Egypt
Source: BMC Med Educ. 2025 Jan 21;25:99. doi: 10.1186/s12909-025-06678-x (PMC11749324; doi:10.1186/s12909-025-06678-x)
Supplement: Supplementary file 1 — Supplementary Material 1. [file 12909_2025_6678_MOESM1_ESM.docx]

**“Prevalence of self‐medication among medical students Tanta University questionnaire”**

**Sociodemographic data Section**

1. Sex : 1  Male 2 Female
2. Age in years:..........
3. Grade:

| 1 first year | 2 second year | 3 third year |
| --- | --- | --- |
| 4 fourth year | 5 fifth year |  |

1. Marital status:

| 1 Single | 2 Engaged | 3 Married |
| --- | --- | --- |

1. Residence:

| 1 Rural | 2 Urban |
| --- | --- |

1. Family income:

| 1 Not enough | 2 Just enough | 3 Enough and saving |
| --- | --- | --- |

1. Father’s education:

| 1 Illiterate | 2 Basic education (primary, preparatory) | 3 Secondary education |
| --- | --- | --- |
| 4 High education |  |  |

1. Mother’s education:

| 1 Illiterate | 2 Basic education (primary, preparatory) | 3 Sea education |
| --- | --- | --- |
| 4 High education |  |  |

1. Total House members:.........

# Self‐Medication habits

**Now let me define what is self‐medication?**

**Self‐medication is the use of medicinal products by the consumer which is not prescribed by the physician. It includes the use of a wide range of complementary and alternative medicine such as herbal medicines (herbs or herbal preparations), nutritional supplements, traditional products, and home remedies.**

**From now on to the next questions, your answers should be based on last 3 months experience, unless I specify the different time period.**

1. Have you done self‐medication in last three months?

**If answer to question 10 is yes, then solve question 11. If not, then go to question 19**

- 1 Yes
- 2 No

1. What kind of self-medication do you use?

**You can select multiple choices by crossing (×) the appropriate boxes below.**

| A Complementary and alternative medicine such as herbal medicines (herbs or herbal preparations) | B Nutritional supplements |
| --- | --- |
| C Home remedies (ginger, mint...etc.) | D pharmaceutical products |

1. If you take pharmaceutical products, what pattern is it?

| A Analgesics | B Antipyretics | Specify: |
| --- | --- | --- |
| C Hypnotics | D Antihistamines |  |
| E NSADs | F others |  |

1. What was your reason for self‐medication**?**

**You can select multiple choices by crossing (×) the appropriate boxes below.**

| a Medical knowledge from self experience and studies | g Saves time |
| --- | --- |
| b Doctor / clinic far from home |  |
| c High fees of doctor | h I have old prescription |
| d Doctor is busy with many patients | i I have medicines of family members |
| e No trust in doctor | j Pharmacist advice |
| f Other. Explain |  |

1. For which disease you have taken self‐medication in last 3 months?

**You can select multiple choices by crossing (×) the appropriate boxes below.**

| 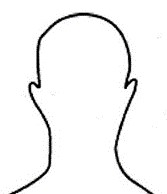 | A Headache | G Eye infection | **Medication name:** |
| --- | --- | --- | --- |
|  | B Dandruff | H Running nose |  |
|  | C Hair fall | I Ear pain |  |
|  | D Faints | J Mouth ulcer |  |
|  | E Epilepsy | K Dental pain |  |
|  | F Migraine | L Cough |  |
|  | M Other. Specify |  |  |
|  | |  |  |
| 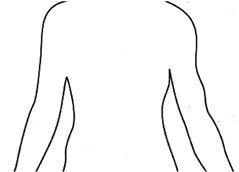 | N Difficulty in swallowing | V Dysentery |  |
|  | O Acidity | W Rash |  |
|  | P Vomiting | X Fever |  |
|  | Q Nausea | Y Skin disease on open areas |  |
|  | R Asthma | Z Diabetes |  |
|  | S Diarrhoea | Aa Hypertension |  |
|  | T Body pain. Specify |  |  |
|  | U Other. Specify |  |  |
|  | |  |  |
| 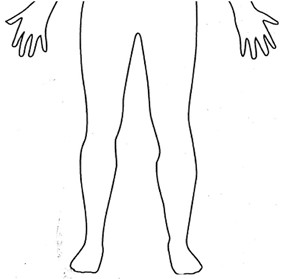 | Ab Pain in joints | Ai Sexually transmitted disease STD |  |
|  | Ac Arthritis | Aj Skin disease in covered areas |  |
|  | Ad Muscle pain | Ak Impotency |  |
|  | Ae Varicose veins | Al Urination problems |  |
|  | Af Wounds | Am Menstrual problems |  |
|  | Ag Genital infection | An Birth control |  |
|  | Ah Other. Specify |  |  |

1. What do you consider while selecting the drug for self‐medication?

**You can select multiple choices by crossing (×) the appropriate boxes below.**

| A Price  B Pharmaceutical company |
| --- |
| C The commonest used medication |
| D Form of medicine ( tablets / syrup / injection...etc. ) |

| 1. From where do you hear about the medication?   **You can select multiple choices by crossing (×) the appropriate boxes below.** | | | |
| --- | --- | --- | --- |
|  | A Recommended by pharmacist | D Old prescription of doctor |  |
|  | B Used by peers – friends / family | E Advertisement |  |
|  | C My previous experience ( medical studies ) | F Other. Explain |  |
|  | | | |

1. Where do you obtain your drugs for self‐medication?

**You can select multiple choices by crossing (×) the appropriate boxes below.**

| A Pharmacy shop | D Online shopping |
| --- | --- |
| B Primary health care centre |  |
| C Friends / family | E Other. Explain |

1. Have you ever experienced side effects with self‐medication?

| 2 No | 1 Yes. Explain ________________________________________________ |
| --- | --- |

**If answer to question 17 is yes, then solve question 18. If not, then go to question 19.**

| 1. What did you do for the side effects you experienced? **You can select multiple choices by crossing (×) the appropriate boxes below.** | | | |
| --- | --- | --- | --- |
|  | A Go to private doctor | D Go to pharmacist |  |
|  | B Go to primary health care centre | E Stop taking medication |  |
|  | C Other. Explain | |  |
|  | | | |

1. Are you taking self‐medication for any chronic disease? **(Chronic disease in this study is defined as a disease lasting three months or longer.)**

| 2 No | 1 Yes |
| --- | --- |

**If answer to question 18 is yes, then solve question 20. If not, then go to the next section of questionnaire on antibiotics.**

| 1. How long you have been taking self‐medication for any chronic disease? | | | |
| --- | --- | --- | --- |
|  | Name of disease: | Time period in months: |  |
|  |  |  |  |
|  |  |  |  |
|  |  |  |  |

# Self‐Medication habits with Antibiotics

1. Have you ever self‐medicated yourself with antibiotics?

| 2 No | 1 Yes |
| --- | --- |

**If answer to question 19 is yes, then go to question 20 or else go to the next section on Health insurance.**

1. How did you know the dosage of antibiotic?

**You can select multiple choices by crossing (×) the appropriate boxes below.**

| A By checking the prescribing information | E Consulting pharmacist | H Internet |
| --- | --- | --- |
| B Consulting doctor | F Consulting peers – friends / family | I Previous self medical knowledge |
| C Consulting primary health care centre | G Advertisements |  |
| D Other. Explain |  |  |

1. When did you stop taking antibiotics**?**

**You can select multiple choices by crossing (×) the appropriate boxes below.**

| A After a few days regardless of the outcome | C After symptoms disappeared | D After antibiotic got over |
| --- | --- | --- |
| B After complete course of antibiotic | | |

# Health insurance

1. Do you have health insurance? Do you use it?

| 1 Yes, I use it | 3 Yes, I don’t use it |
| --- | --- |
| 2 No, I don’t have |  |

1. Does it cover over the counter drugs?

| 1 Yes | 2 No |
| --- | --- |
